# Digoxin versus placebo, no intervention, or other medical interventions for atrial fibrillation and atrial flutter: a protocol for a systematic review with meta-analysis and Trial Sequential Analysis

**DOI:** 10.1186/s13643-017-0470-2

**Published:** 2017-04-05

**Authors:** Naqash J. Sethi, Sanam Safi, Joshua Feinberg, Emil E. Nielsen, Christian Gluud, Janus C. Jakobsen

**Affiliations:** 1 Copenhagen Trial Unit, Centre for Clinical Intervention Research, Department 7812, Rigshospitalet, Copenhagen University Hospital, Copenhagen, Denmark; 2 The Cochrane Hepato-Biliary Group, Copenhagen Trial Unit, Centre for Clinical Intervention Research, Department 7812, Rigshospitalet, Copenhagen University Hospital, Copenhagen, Denmark; 3 Department of Cardiology, Holbæk Hospital, Holbæk, Denmark

**Keywords:** Atrial fibrillation, Atrial flutter, Digoxin, Medical therapy, Systematic review, Meta-analysis, Trial Sequential Analysis

## Abstract

**Background:**

Atrial fibrillation is the most common arrhythmia of the heart with a prevalence of approximately 2% in the western world. Atrial flutter, another arrhythmia, occurs less often with an incidence of approximately 200,000 new patients per year in the USA. Patients with atrial fibrillation and atrial flutter have an increased risk of death and morbidities. In the management of atrial fibrillation and atrial flutter, it is often necessary to use medical interventions to lower the heart rate. Lowering the heart rate may theoretically prevent the development of heart failure and tachycardia-mediated cardiomyopathy. The evidence on the benefits and harms of digoxin compared with placebo or with other medical interventions is unclear. This protocol for a systematic review aims at identifying the beneficial and harmful effects of digoxin compared with placebo, no intervention, or with other medical interventions for atrial fibrillation and atrial flutter.

**Methods:**

This protocol for a systematic review was conducted following the recommendations of Cochrane and the eight-step assessment procedure suggested by Jakobsen and colleagues. We plan to include all relevant randomised clinical trials comparing digoxin with placebo, no intervention, or with other medical interventions. We plan to search the Cochrane Central Register of Controlled Trials (CENTRAL), MEDLINE, EMBASE, LILACS, Science Citation Index Expanded on Web of Science, and BIOSIS to identify relevant trials. Any eligible trial will be assessed and classified as either at high risk of bias or low risk of bias, and our primary conclusions will be based on trials with low risk of bias. We will perform our meta-analyses of the extracted data using Review Manager 5.3 and Trial Sequential Analysis ver. 0.9.5.5 beta. For both our primary and secondary outcomes, we will create a ‘Summary of Findings’ table based on GRADE assessments of the quality of the evidence.

**Discussion:**

The results of this systematic review have the potential to benefit millions of patients worldwide as well as healthcare economy.

**Systematic review registration:**

PROSPERO CRD42016052935

**Electronic supplementary material:**

The online version of this article (doi:10.1186/s13643-017-0470-2) contains supplementary material, which is available to authorized users.

## Background

Atrial fibrillation is the most common arrhythmia of the heart with a prevalence of approximately 2% in the western world [1, 2]. Atrial flutter, another arrhythmia, occurs less often with an incidence of approximately 200,000 new patients per year in the USA [3]. The prevalence of both atrial fibrillation and atrial flutter are increasing possibly because of a greater life expectancy in the general population, an increased prevalence of risk factors for atrial fibrillation and atrial flutter, and an improved ability to suspect and diagnose the arrhythmias [1, 4, 5]. Atrial fibrillation and atrial flutter are associated with an increased risk of death and morbidities [6–12]. The risks of both cerebral stroke and heart failure are increased nearly fivefold in patients with atrial fibrillation and atrial flutter, and an estimated 20% of every stroke may be due to atrial fibrillation [6–11]. Atrial fibrillation and atrial flutter also have a significant impact on healthcare costs and account for approximately 1% of the National Health Service budget in the UK and approximately 26 billion dollars of annual expenses in the USA [13, 14].

## Definition and classification

The atriums of the heart receive blood returning from the body and pump it further ahead to the ventricles. Atrial fibrillation and atrial flutter are defined as abnormal heart rhythms that arise from improper electrical activity of the heart which lead to ineffective mechanical contraction [15–17]. The ineffective mechanical contraction stresses the muscle cells of the heart which over time may cause heart failure [18, 19]. Persistent rapid rates can also cause or worsen a tachycardia-mediated cardiomyopathy [20].

Atrial fibrillation and atrial flutter can be asymptomatic or lead to symptoms such as palpitations, dyspnoea, and dizziness [21]. Atrial fibrillation may be diagnosed using an electrocardiogram as (1) irregular R-R intervals (when atrioventricular conduction is present), (2) absence of distinct repeating P-waves, and (3) irregular atrial activity [16, 17]. Atrial flutter may be diagnosed using an electrocardiogram as characteristic flutter waves (F-waves) at a regular atrial rate of 250 to 350 beats per minute. The flutter waves may resemble P-waves or have a ‘saw-tooth’ shape [22].

Atrial fibrillation may either be non-valvular or valvular, where the latter form is characterised by rheumatic mitral stenosis, mechanical heart valve, tissue heart valve, or mitral valve repair [1]. However, the definition of the terms non-valvular and valvular lacks consistency in both trials and guidelines [23, 24]. A paper has proposed a new term ‘mechanical and rheumatic mitral valvular atrial fibrillation’, as the authors report that only mechanical valves and mitral stenosis have special needs in regard to antithrombotic treatment [24].

The development of atrial fibrillation is associated with various risk factors, e.g. ageing, obesity, smoking, hypertension, diabetes, and other cardiac diseases (valvular or other structural heart diseases) [17, 25]. The development of atrial flutter is presumably associated with prolonged PR interval on an electrocardiogram and some of the same risk factors as atrial fibrillation [8]. However, it has not been demonstrated that atrial flutter is associated with either obesity, diabetes, hypertension, or valvular heart disease [8]. Both atrial fibrillation and atrial flutter may also occur in patients with no risk factors (so-called lone atrial fibrillation or lone atrial flutter) [3].

Based on the duration of the arrhythmia, atrial fibrillation may be divided into five different forms [15–17]:

- Recent-onset atrial fibrillation
- Paroxysmal atrial fibrillation
- Persistent atrial fibrillation
- Long-standing persistent atrial fibrillation
- Permanent atrial fibrillation

Based on the re-entrant circuit, atrial flutter may be divided into two different forms:

- Typical atrial flutter is a macro-reentrant atrial tachycardia that can be subdivided based on the rotation of the circuit to counterclockwise atrial flutter (90% of patients) or clockwise atrial flutter (10% of patients) [26].
- Atypical atrial flutter is defined as any atrial tachycardia with an electrocardiogram pattern of continuous undulation of the atrial complex, different from typical atrial flutter, at a rate of ≥240 beats per minute [26].

## Pathophysiology

The pathogenesis of atrial fibrillation is thought to be an interaction between a trigger for initiation and an abnormal tissue substrate for maintenance [25].

The trigger for initiation is often a rapidly firing focus most often located in the left atrium and the proximal parts of the pulmonary veins [27]. The abnormal tissue substrate for maintenance is often a result of an underlying heart disease like coronary heart disease, valvular heart disease, cardiomyopathies, or heart failure [16]. The pathogenesis of the abnormal tissue substrate is induced by inflammation [28], fibrosis [29], or hypertrophy [30].

Electric remodelling, such as refractory period shortening, occurs after a period of continuous atrial fibrillation that further facilitates atrial fibrillation, i.e. atrial fibrillation leads to atrial fibrillation [30, 31]. Nevertheless, the electric remodelling is often reversible if sinus rhythm is restored, though it can become permanent if atrial fibrillation persists [31].

Atrial flutter is classified as a macro-reentrant tachycardia. The macro-reentrant tachycardia occurs when an electrical impulse recurrently moves in a self-perpetuating circuit within the heart, rather than moving from one end of the heart to the other and terminating [26].

## Antithrombotic treatment

As mentioned (see the ‘Background’ section), the risk of stroke is increased nearly fivefold in patients with atrial fibrillation and atrial flutter [10]. Antithrombotic treatment is necessary to reduce the risk of stroke in high-risk patients with atrial fibrillation and atrial flutter, regardless of the management strategy of the heart disease [16]. The risk of stroke in patients with atrial fibrillation and atrial flutter can be estimated by the CHA2DS2-VASc score [32], while the risk of bleeding can be estimated by the HAS-BLED score [33]. Combined, these scores may help the physician determine the patient’s need for antithrombotic treatment [16].

Antithrombotic drugs aim at reducing the formation of thrombi by affecting different clotting processes. Depending on the mechanism, the drugs are divided into either anticoagulants or antiplatelet drugs. The classification, mechanism, and examples of anticoagulants and antiplatelet drugs are summarised in Table 1.

**Table 1:** The classification, mechanism, and examples of anticoagulants and antiplatelet drugs

Class	Mechanism	Examples
Anticoagulants	Affect the coagulation cascade to prevent blood coagulation [112]	Vitamin K-dependent antagonists •Warfarin Non-vitamin K-dependent antagonists •Dabigatran •Rivaroxaban •Apixaban •Edoxaban Heparin •Unfractionated heparin •Low-molecular-weight heparin
Antiplatelet drugs	Theoretically affect the migration and aggregation of platelets, consequently aiming at inhibiting thrombus formation [112]	•Aspirin •Clopidogrel •Prasugrel •Ticagrelor •Cangrelor •Abciximab •Eptifibatid •Dipyridamole

The comparative efficacy and safety between anticoagulants and antiplatelet drugs has been assessed. Two systematic reviews have shown that warfarin (vitamin K-dependent antagonist) and apixaban (non-vitamin K-dependent antagonist) are both superior to antiplatelet drugs for preventing stroke, with a comparable rate of major bleeding and intracranial haemorrhage [34, 35].

The comparative benefits and harms between warfarin and non-vitamin K-dependent antagonists has been assessed. Ruff et al. showed in a systematic review that the non-vitamin K-dependent antagonists compared with warfarin significantly reduced the risk of all-cause mortality by 10%, stroke by 19%, and intracranial haemorrhage by 52% [36]. However, the risk of gastrointestinal bleeding was increased by 25% by the non-vitamin K-dependent antagonists [36].

## Description of the intervention

As mentioned (see the ‘Background’ section), atrial fibrillation and atrial flutter cause the atriums and ventricles to beat rapidly and irregularly which stresses the muscle cells of the heart. An intervention to control the heart rate, mainly digoxin, beta blockers, non-dihydropyridine calcium channel blockers, amiodarone, or sotalol, is therefore often necessary to reduce the heart rate and consequently prevent excessive tachycardia and limit symptoms in nearly all patients with atrial fibrillation and atrial flutter [16, 22]. Lowering the heart rate might also theoretically prevent the development of heart failure and tachycardia-mediated cardiomyopathy [18, 20, 37].

The drugs used as treatment for atrial fibrillation and atrial flutter are classified according to two different classifications: the Vaughan Williams classification and the Sicilian Gambit classification.

- The Vaughan Williams classification classifies the drugs in five different classes according to their general effect [38]. The Vaughan Williams classification is summarised in Table 2.

**Table 2:** The Vaughan Williams classification

Class	Mechanism	Examples
Ia	Na^+^ channel block (moderate)	•Quinidine •Ajmaline •Procainamide •Disopyramide
Ib	Na^+^ channel block (weak)	•Lidocaine •Phenytoin •Mexiletine •Tocainide
Ic	Na^+^ channel block (strong)	•Flecainide •Propafenone •Encainide •Moricizine
II	Beta-blocker	•Propranolol •Carvedilol •Esmolol •Timolol •Metoprolol •Atenolol •Bisoprolol •Nebivolol
III	K^+^ channel blocker	•Amiodarone •Dronedarone •Sotalol •Ibutilide •Dofetilide •Vernakalant
IV	Ca^2+^ channel blocker	•Verapamil •Diltiazem
V	Variable	•Adenosine •Digoxin •Magnesium sulphate
- The Sicilian Gambit classification places a greater focus on the underlying mechanism of the drugs and classifies each drug according to the effects on bio-cellular channels, receptors, and pumps. We will not describe this classification in detail but refer to the work by the European Society of Cardiology [39].

We will in this systematic review use the Vaughan Williams classification as it is the most commonly used classification.

### Digoxin

Digoxin (Vaughan Williams class V) is thought to increase the contractility of the heart (inotropic effect) and decreases the heart rate (chronotropic effect). The contractility of the heart is increased by inhibiting the Na^+^/K^+^ ATPase. The inhibited Na^+^/K^+^ ATPase results in a higher concentration of intracellular calcium which leads to an increase in the left ventricular systolic function [40]. The heart rate is decreased by inducing vagal activation which presumably slows down the conduction of the atrioventricular node, consequently lowering the heart rate [41].

The Atrial Fibrillation Follow-up Investigation of Rhythm Management (AFFIRM) trial showed that digoxin (used without beta-blockers or non-dihydropyridine calcium channel blockers) achieved rate control (rest ≤80 beats/min) in 58% of the patients given digoxin as first therapy [42].

### Other medical interventions

Beta-blockers (Vaughan Williams class II) block the sympathetic activity in the atrioventricular node, consequently decreasing the heart rate [41]. The different beta-blockers are illustrated in Table 2. Beta-blocker monotherapy is often the recommended first-line therapy for heart rate control in atrial fibrillation and atrial flutter [43]. The AFFIRM trial showed that beta-blockers (with or without digoxin) achieved rate control (rest ≤80 beats/min) in 70% of the patients given beta-blockers as first therapy [42].

Non-dihydropyridine calcium channel blockers (Vaughan Williams class IV) slow the atrioventricular node conduction by blocking calcium channels and thus lower the heart rate [41]. The main non-dihydropyridine calcium channel blockers are illustrated in Table 2. Non-dihydropyridine calcium channel blockers are reasonable in controlling the heart rate [44] but are not recommended in patients with concomitant heart failure because of their negative inotropic effects [45]. The AFFIRM trial showed that non-dihydropyridine calcium channel blockers (with or without digoxin achieved rate control (rest ≤80 beats/min) in 54% of the patients given non-dihydropyridine calcium channel blockers as first therapy [42].

Amiodarone (Vaughan Williams class III) may also control the heart rate, as it exhibits beta and calcium channel blockade in addition to its antiarrhythmic activity. However, amiodarone has extensive non-cardiac adverse events and is only used if other rate control drugs are not effective enough, not well tolerated, or contraindicated [16].

Sotalol (Vaughan Williams class III) may also control the heart rate, as it exhibits beta blockade in addition to its antiarrhythmic activity. However, sotalol may induce life-threatening arrhythmias such as torsades de pointes [46]. Accordingly, it is seldom used.

## Why is it important to do this review?

Digoxin is widely used for heart rate control in patients with atrial fibrillation and atrial flutter. Guidelines recommend using digoxin as the primary drug for heart rate control in patients with atrial fibrillation and atrial flutter who have concomitant heart failure and reduced ejection fraction [16, 43, 47]. Digoxin is also recommended for acute heart rate control in patients with preserved ejection fraction [16, 43, 47]. Nevertheless, a report from 2014 has shown a dramatic decrease in overall digoxin treatment visits in the USA, from 12.9 million visits in 1997 to 1.87 million visits in 2012 [48].

According to the newest guideline by the European Society of Cardiology, there are no head-to-head randomised trials of digoxin versus other medical interventions in relation to heart rate control in patients with atrial fibrillation or atrial flutter [43]. However, our group found several randomised trials by performing a preliminary search in Medical Literature Analysis and Retrieval System Online (MEDLINE) [49–52]. Siu et al. compared diltiazem, digoxin, and amiodarone and showed superior rate control in the diltiazem group [49]. Joseph et al. compared sotalol, amiodarone, and digoxin and showed superior rate control in the sotalol group [50]. Tisdale et al. compared diltiazem with digoxin and showed superior rate control in the diltiazem group [51]. Tse et al. compared digoxin with amiodarone and showed similar rate control efficacy in the compared groups [52].

During recent years, systematic reviews of observational studies have compared digoxin versus no digoxin (the latter participants usually receiving some other treatment for heart rate control) in patients with atrial fibrillation or atrial flutter and showed that digoxin seemed to increase the risk of all-cause mortality regardless of concomitant heart failure [53–56]. A systematic review of both observational studies and randomised clinical trials showed similar findings on all-cause mortality when assessing the observational studies. However, they did not show any difference on all-cause mortality when assessing the randomised clinical trials. They reported that the observed association between digoxin and mortality in observational studies might be a result of confounding that cannot be mitigated by statistical adjustment [57].

In 2014, a systematic review of randomised trials assessed the drugs used for heart rate control and included 16 randomised clinical trials. Eight of them were head-to-head trials comparing digoxin with other medical heart rate control interventions. The authors reported that the data were inconclusive as to whether any one drug was safer or more effective than the others. However, the review authors did not use any method to deal with risks of random errors [58]. Therefore, it is still unclear whether digoxin offers more benefits and less harms compared with placebo, no intervention, or with other medical interventions in controlling the heart rate. No former systematic review comparing digoxin with placebo, no intervention, or with other medical interventions for atrial fibrillation and atrial flutter has taken account both risks of systematic errors and risks of random errors (Cochrane methodology, Trial Sequential Analysis, and the Grades of Recommendation, Assessment, Development, and Evaluation (GRADE) assessment). In the present systematic review, we will collect and present current evidence of digoxin versus placebo, no intervention, or with other medical interventions for atrial fibrillation and atrial flutter.

## Objectives

This review has two objectives:

1. To assess the beneficial and harmful effects of digoxin compared with placebo or no intervention for atrial fibrillation and atrial flutter
2. To assess the beneficial and harmful effects of digoxin compared with other medical interventions for atrial fibrillation and atrial flutter

## Methods

This systematic review protocol has been developed based on Preferred Reporting Items for Systematic Reviews and Meta-Analysis Protocols (PRISMA-P) guidelines for reporting systematic reviews evaluating healthcare interventions [59, 60]. A PRISMA-P checklist file is attached (Additional file 1).

## Criteria for considering studies for this review

### Types of studies

Randomised clinical trials irrespective of trial design, setting, publication status, publication year, and language. We will not specifically search for non-randomised studies. However, if we during our literature searches identify non-randomised studies (quasi-randomised studies or observational studies) with adequate reports of harmful effects, then we will narratively report these results. By focusing on randomised clinical trials, we are aware that the present review will be biased towards focusing on benefits and less on harms. Trials that only include a subset of eligible participants will only be included if (1) separate data on the eligible participants are available or (2) the majority of participants are eligible. We will document difficult decisions in the review, and sensitivity analyses will assess the impact of these decisions on the findings of the review. Randomised clinical trials will be included irrespective of the reporting of one of the outcomes in this protocol.

### Types of participants

Patients with atrial fibrillation or atrial flutter. We will accept the definitions used by the trialists. Patients will be included irrespective of age, sex, and comorbidities.

### Types of interventions

We will include four types of trials:

- Digoxin compared with placebo
- Digoxin compared with no intervention
- Digoxin added to a co-intervention compared with a similar co-intervention
- Digoxin compared with any type of medical intervention other than digoxin (e.g. beta-blockers, non-dihydropyridine calcium channel blockers, amiodarone, or sotalol)

We will as experimental intervention accept digoxin irrespective of dose, route of administration, and duration of administration.

All control interventions will be included irrespective of dose, route of administration, and duration.

We will accept any type of co-intervention when such co-intervention is intended to be delivered similar to the experimental and the control group (i.e. we will exclude trials that, e.g. add a beta-blocker to the experimental group and not to the control group).

### Types of outcome measures

We will for all outcomes, beside heart rate control and conversion to sinus rhythm, use the trial results at maximal follow-up. We will for heart rate control and conversion to sinus rhythm use the trial results reported within 48 h to assess the acute effects of digoxin versus placebo, no intervention, or other medical interventions in patients with atrial fibrillation or atrial flutter. We will, additionally, conduct sensitivity analyses excluding all trials with short (less than 12 months) or long (more than 24 months) follow-up in the primary meta-analyses of the outcome results at maximal follow-up (i.e. all-cause mortality, serious adverse events, quality of life, heart failure, and stroke).

#### Primary outcomes

1. All-cause mortality.
2. Serious adverse events. We will define a serious adverse event as any untoward medical occurrence that resulted in death, was life-threatening, required hospitalisation or prolongation of existing hospitalisation, and resulted in persistent or significant disability or jeopardised the patient [61].
3. Quality of life measured on any valid scale.

#### Secondary outcomes

1. Heart failure (as defined by the trialists)
2. Stroke (as defined by the trialists)
3. Heart rate control
4. Conversion to sinus rhythm (as defined by the trialists)

All outcomes, except quality of life and heart rate control, will be analysed as proportions of participants in each group.

## Search methods for identification of studies

### Electronic searches

We will search the Cochrane Central Register of Controlled Trials (CENTRAL), MEDLINE, EMBASE, LILACS, Science Citation Index Expanded on Web of Science, and BIOSIS in order to identify relevant trials. The preliminary search strategy for MEDLINE (Ovid) is given in Additional file 2.

We will search all databases from their inception to the present, and we will impose no restriction on language of publication or publication status.

### Searching other resources

The reference lists of relevant publications will be checked for any unidentified randomised trials. We will contact authors of included studies, and major pharmaceutical companies, by email asking for unpublished randomised trials. Further, we will search for ongoing trials on:

- ClinicalTrials.gov (www.clinicaltrials.gov);
- Google Scholar (https://scholar.google.dk/);
- The Turning Research into Practice (TRIP) Database (https://ww.tripdatabase.com/);
- European Medicines Agency (EMA) (http://www.ema.europa.eu/ema/);
- United States Food and Drug Administration (FDA) (www.fda.gov);
- China Food and Drug Administration (CFDA) (http://eng.sfda.gov.cn/WS03/CL0755/);
- Medicines and Healthcare products Regulatory Agency
- (https://www.gov.uk/government/organisations/medicines-and-healthcare-products-regulatory-agency);
- The World Health Organization (WHO) International Clinical Trials Registry Platform (ICTRP) search portal (http://apps.who.int/trialsearch/).

Additionally, we will hand search conference abstracts from cardiology conferences for relevant trials.

We will also include unpublished and grey literature trials if we identify these, and assess relevant retraction statements and errata for included studies.

## Data collection and analysis

We will perform the review following the recommendations of Cochrane [62]. The analyses will be performed using Review Manager 5.3 [63] and Trial Sequential Analysis ver. 0.0.5.5 beta [64]. In case of Review Manager statistical software not being sufficient, we will use STATA 14 [65].

### Selection of studies

Two authors (NJS and SS) will independently screen titles and abstracts. We will retrieve all relevant full-text study reports/publications, and two review authors (NJS and SS) will independently screen the full-text and identify and record reasons for exclusion of the ineligible studies. We will resolve any disagreement through discussion, or if required, we will consult a third person (JCJ). Trial selection will be displayed in an adapted flow diagram as per the Preferred Reporting Items for Systematic Reviews and Meta-Analyses (PRISMA) statement [66].

### Data extraction and management

Four authors (NJS, SS, EEN, and JF) will extract data independently from the included trials. Disagreements will be resolved by discussion with a fifth author (JCJ). We will assess duplicate publications and companion papers of a trial together in order to evaluate all available data simultaneously (maximise data extraction, correct bias assessment). We will contact the trial authors by email to specify any additional data, which may not have been reported sufficiently or at all in the publication.

#### Trial characteristics

Bias risk components (as defined below); trial design (parallel, factorial, or crossover); number of intervention arms; length of follow-up; estimation of sample size; inclusion and exclusion criteria.

#### Participants’ characteristics and diagnosis

Number of randomised participants; number of analysed participants; number of participants lost to follow-up/withdrawals/crossover; compliance with medication; age range (mean or median) and sex ratio; type of arrhythmia (atrial fibrillation or atrial flutter); baseline numbers of cardiovascular risk factors (i.e. diabetes mellitus, hypertension, hyperlipidaemia, or smoking); baseline number of participants with heart failure; baseline number of participants with valvular heart disease; baseline number of participants with previous myocardial infarction; baseline number of participants with previous revascularisation; and baseline number of participants with previous angina.

#### Intervention characteristics

Dose of intervention, duration of intervention, and mode of administration.

#### Control characteristics

Type of control intervention, dose of intervention, duration of intervention, and mode of administration.

#### Co-intervention characteristics

Type of co-intervention, dose of co-intervention, duration of co-intervention, and mode of administration.

#### Outcomes

All outcomes listed above will be extracted from each randomised clinical trial, and we will identify if outcomes are incomplete or selectively reported according to the criteria described later in ‘incomplete outcome data’ bias domain and ‘selective outcome reporting’ bias domain.

#### Notes

Funding of the trial and notable conflicts of interest of trial authors will be extracted, if available.

We will note in the ‘Characteristics of included studies’ table if outcome data were not reported in a usable way. Two review authors (NJS and SS) will independently transfer data into the Review Manager file [63]. Disagreements will be resolved through discussion, or if required, we will consult with a third author (JCJ).

### Assessment of risk of bias in included studies

We will use the instructions given in the Cochrane Handbook for Systematic Reviews of Interventions [62] in our evaluation of the methodology and hence the risk of bias of the included trials. We will evaluate the methodology in respect of:

- Random sequence generation
- Allocation concealment
- Blinding of participants and personnel
- Blinding of outcome assessment
- Incomplete outcome data
- Selective outcome reporting
- Other risks of bias
- Overall risk of bias

These domains enable classification of randomised trials with low risk of bias and high risk of bias. The latter trials tend to overestimate positive intervention effects (benefits) and underestimate negative effects (harms) [67–73].

We will classify the trials according to the following criteria:

#### Random sequence generation

- Low risk: If sequence generation was achieved using computer random number generator or a random number table. Drawing lots, tossing a coin, shuffling cards, and throwing dice were also considered adequate if performed by an independent adjudicator.
- Unclear risk: If the method of randomisation was not specified, but the trial was still presented as being randomised.
- High risk: If the allocation sequence is not randomised or only quasi-randomised. These trials will be excluded.

#### Allocation concealment

- Low risk: If the allocation of patients was performed by a central independent unit, on-site locked computer, identical-looking numbered sealed envelopes, drug bottles, or containers prepared by an independent pharmacist or investigator.
- Uncertain risk: If the trial was classified as randomised but the allocation concealment process was not described.
- High risk: If the allocation sequence was familiar to the investigators who assigned participants.

#### Blinding of participants and personnel

- Low risk: If the participants and the personnel were blinded to intervention allocation and this was described.
- Uncertain risk: If the procedure of blinding was insufficiently described.
- High risk: If blinding of participants and the personnel was not performed.

#### Blinding of outcome assessment

- Low risk of bias: If it was mentioned that outcome assessors were blinded and this was described.
- Uncertain risk of bias: If it was not mentioned if the outcome assessors in the trial were blinded, or the extent of blinding was insufficiently described.
- High risk of bias: If no blinding or incomplete blinding of outcome assessors was performed.

#### Incomplete outcome data

- Low risk of bias: If missing data were unlikely to make treatment effects depart from plausible values. This could either be (1) there were no drop-outs or withdrawals for all outcomes or (2) the numbers and reasons for the withdrawals and drop-outs for all outcomes were clearly stated and could be described as being similar to both groups. Generally, the trial is judged as at a low risk of bias due to incomplete outcome data if drop-outs are less than 5%. However, the 5% cut-off is not definitive.
- Uncertain risk of bias: If there was insufficient information to assess whether missing data were likely to induce bias on the results.
- High risk of bias: If the results were likely to be biased due to missing data either because the pattern of drop-outs could be described as being different in the two intervention groups or the trial used improper methods in dealing with the missing data (e.g. last observation carried forward).

#### Selective outcome reporting

- Low risk of bias: If a protocol was published before or at the time the trial was begun and the outcomes specified in the protocol were reported on. If there is no protocol or the protocol was published after the trial has begun, reporting of all-cause mortality and serious adverse events will grant the trial a grade of low risk of bias.
- Uncertain risk of bias: If no protocol was published and the outcomes all-cause mortality and serious adverse events were not reported on.
- High risk of bias: If the outcomes in the protocol were not reported on.

#### Other risks of bias

- Low risk of bias: If the trial appears to be free of other components (for example, academic bias or for-profit bias) that could put it at risk of bias.
- Unclear risk of bias: If the trial may or may not be free of other components that could put it at risk of bias.
- High risk of bias: If there are other factors in the trial that could put it at risk of bias (for example, authors conducted trials on the same topic, for-profit bias, etc.).

#### Overall risk of bias

- Low risk of bias: The trial will be classified as overall ‘low risk of bias’ only if all of the bias domains described in the above paragraphs are classified as ‘low risk of bias’.
- High risk of bias: The trial will be classified ‘high risk of bias’ if any of the bias risk domains described in the above are classified as ‘unclear’ or ‘high risk of bias’.

We will assess the domains ‘blinding of outcome assessment’, ‘incomplete outcome data’, and ‘selective outcome reporting’ for each outcome result. Thus, we will assess the bias risk for each outcome assessed in addition to each trial. Our primary conclusions will be based on the results of both our primary and secondary outcome results with overall low risk of bias. If no results with overall low risk of bias are found, we will base our primary conclusions on the outcome results irrespective of overall risk of bias. We will present our primary conclusions in the ‘Summary of Findings’ table.

#### Differences between the protocol and the review

We will conduct the review according to this published protocol and report any deviations from it in the ‘Differences between the protocol and the review’ section of the systematic review.

#### Measures of treatment effect

##### Dichotomous outcomes

We will calculate risk ratios (RRs) with 95% confidence interval (CI) for dichotomous outcomes. We will also report Trial Sequential Analysis-adjusted CIs (see paragraphs below).

##### Continuous outcomes

We will calculate the mean differences (MDs) and if necessary, as a hypothesis generating analysis, the standardised mean difference (SMD) with 95% CI for continuous outcomes. We will also report Trial Sequential Analysis-adjusted CIs (see paragraphs below).

#### Dealing with missing data

We will, as first option, contact all trial authors to obtain any relevant missing information and data (i.e. for data extraction and for assessment of risk of bias, as specified above).

##### Dichotomous outcomes

We will not impute missing values for any outcomes in our primary analysis. In two of our sensitivity analyses (see paragraph below), we will impute data.

##### Continuous outcomes

We will primarily analyse scores assessed at single time points. If only change from baseline scores are reported, we will analyse the results together with follow-up scores [62]. If standard deviations (SDs) are not reported, we will calculate the SDs using trial data, if possible. We will not use intention-to-treat data if the original report did not contain such data. We will not impute missing values for any outcomes in our primary analysis. In our sensitivity analysis (see paragraph below) for continuous outcomes, we will impute data.

#### Assessment of heterogeneity

We will primarily investigate forest plots to visually assess any sign of heterogeneity. We will secondly assess the presence of statistical heterogeneity by chi^2^ test (threshold *P* < 0.10) and measure the quantities of heterogeneity by the *I* ^2^-statistic [74, 75].

We will follow the recommendations for threshold by the *Cochrane Handbook for Systemic Reviews of Interventions* [62]:

- 0 to 40%: might not be important
- 30 to 60%: may represent moderate heterogeneity
- 50 to 90%: may represent substantial heterogeneity
- 75 to 100%: may represent considerable heterogeneity

We will investigate possible heterogeneity through subgroup analyses. Ultimately, we may decide that a meta-analysis should be avoided [62].

#### Assessment of reporting biases

We will use a funnel plot to assess reporting bias if ten or more trials are included. We will visually inspect funnel plots to assess the risk of bias. We are aware of the limitations of a funnel plot, i.e. a funnel plot assesses bias due to small sample size and asymmetry of a funnel plot is not necessarily caused by reporting bias. From this information, we assess possible reporting bias. For dichotomous outcomes, we will test asymmetry with the Harbord test [76] if *τ* ^2^ is less than 0.1 and with the Rücker test if *τ* ^2^ is more than 0.1. For continuous outcomes, we will use the regression asymmetry test [77] and the adjusted rank correlation [78].

### Unit of analysis issues

We will only include randomised clinical trials. For trials using crossover design, only data from the first period will be included [62, 79]. There will therefore not be any unit of analysis issues. We will not include cluster randomised trials, as these have a high risk of biased results due to confounding [62].

### Data synthesis

#### Meta-analysis

We will undertake this meta-analysis according to the recommendations stated in the *Cochrane Handbook for Systematic Reviews of Interventions* [62], Keus et al. [80], and the eight-step assessment suggested by Jakobsen et al. for better validation of meta-analytic results in systematic reviews [81]. We will use the statistical software Review Manager 5.3 [63] provided by Cochrane to analyse data.

We will assess our intervention effects with both random effects meta-analyses [82] and fixed effects meta-analyses [83]. We will use the more conservative point estimate of the two [81]. The more conservative point estimate is the estimate closest to zero effect. If the two estimates are similar, we will use the estimate with the widest CI. We will conduct sensitivity analyses and subgroup analyses to explore the reasons for substantial statistical heterogeneity (see the ‘Assessment of heterogeneity’ section). We will assess the risk of publication bias in meta-analyses consisting of ten trials or more by visually inspection of forest plots and statistical tests for funnel plot asymmetry (see the ‘Assessment of reporting biases’ section). We adjust our thresholds for statistical significance due to problems with multiplicity (family-wise error rate) by dividing the prespecified *P* value threshold with the value halfway between 1 (no adjustment) and the number of primary or secondary outcome comparisons (Bonferroni adjustment) [81, 84]. We use three primary outcomes, and therefore, we will consider a *P* value of 0.025 or less as the threshold for statistical significance for these outcomes [81]. We use four secondary outcomes, and therefore, we will consider a *P* value of 0.02 or less as threshold for statistical significance for these outcomes [81]. We will use the eight-step procedure to assess if the thresholds for significance are crossed [81]. Our primary conclusion will be based on results with low risk of bias [81].

Where multiple trial intervention groups are reported in a single trial, we will include only the relevant groups. If two comparisons are combined in the same meta-analysis, we will halve the control group to avoid double-counting [81, 85].

Trials with a factorial design will be included. In case of, e.g. a 2 × 2 factorial designed trial, the two groups receiving digoxin will be considered experimental groups, while the two groups receiving other medical rate control interventions will be considered control groups.

If quantitative synthesis is not appropriate, we will report the results in a narrative way.

#### Trial Sequential Analysis

Cumulative meta-analyses are at risk of producing random errors due to sparse data and multiple testing of accumulating data [64, 85–94]. Therefore, Trial Sequential Analysis [64] can be applied to control these risks (http://www.ctu.dk/tsa/) [91]. Similar to a sample size calculation in a randomised clinical trial, Trial Sequential Analysis estimates the required information size (that is the number of participants needed in a meta-analysis to detect or reject a certain intervention effect) in order to minimise random errors [89]. The required information size takes into account the anticipated intervention effect, the variance of the anticipated difference in intervention effects, the acceptable risk of falsely rejecting the null hypothesis (alpha), the acceptable risk of falsely confirming the null hypothesis (beta), and the variance of the intervention effect estimated between trials [81, 89, 95]. We tried to base the anticipated intervention effects on empirical data [81]. However, no suitable empirical data could be found, and instead, we pragmatically hypothesised the anticipated intervention effects. When analysing dichotomous outcomes, we pragmatically anticipated an intervention effect of 15% relative risk reduction. When analysing continuous outcomes, we pragmatically anticipated an intervention effect equal to the mean difference of the observed SD/2 [96].

Trial Sequential Analysis enables testing for significance to be conducted each time a new trial is included in the meta-analysis. On the basis of the required information size, trial sequential monitoring boundaries are constructed. This enables one to determine the statistical inference concerning cumulative meta-analysis that has not yet reached the diversity-adjusted required information size [86, 89].

Firm evidence for benefit or harm may be established if a trial sequential monitoring boundary is crossed before reaching the diversity-adjusted required information size, in which case further trials may turn out to be superfluous. In contrast, if a boundary is not surpassed, one may conclude that it is necessary to continue with further trials before a certain intervention effect can be detected or rejected. Firm evidence for lack of the postulated intervention effect can also be assessed with Trial Sequential Analysis. This occurs when the cumulative Z-score crosses the trial sequential boundaries for futility.

For dichotomous outcomes, we will estimate the required information size based on a relative risk reduction of 15%, the observed proportion of participants with an outcome in the control group, an alpha of 2.5% for our primary outcomes and 2.0% for our secondary outcomes (see the ‘Meta-analysis’ section), a beta of 10%, and diversity as suggested by the trials in the meta-analysis (diversity-adjusted required information size) [81, 89]. Additionally, we will calculate Trial Sequential Analysis-adjusted CIs.

For continuous outcomes, we will estimate the required information size based on a minimal clinically important difference of SD/2, the standard deviation observed in the control group, an alpha of 2.5% for our primary outcomes and 2.0% for our secondary outcomes (see ‘Meta-analysis’), a beta of 10%, and a diversity as suggested by the trials in the meta-analysis (diversity adjusted required information size) [81, 89]. Additionally, we will calculate Trial Sequential Analysis-adjusted CIs.

### Subgroup analysis and investigation of heterogeneity

#### Subgroup analysis

We will perform the following subgroup analyses when analysing the primary outcomes (all-cause mortality, serious adverse event, and quality of life).

1. High risk of bias trials compared to low risk of bias trials
2. Comparison of different types of control interventions
3. Participants with atrial fibrillation compared to participants with atrial flutter
4. Age of participants: 0 to 59 years, 60 to 79 years, and above 80 years
5. Duration of atrial fibrillation: recent-onset atrial fibrillation (as defined by the trialists), paroxysmal atrial fibrillation (less than 7 days of onset), persistent atrial fibrillation (more than 7 days and less than 1 year of onset), and long-standing persistent atrial fibrillation (more than 1 year of onset).
6. Men compared to women
7. Participants with heart failure compared to participants without heart failure
8. Comparison of trials with different medical co-intervention

We will use the formal test for subgroup differences in Review Manager [63].

#### Sensitivity analysis

To assess the potential impact of the missing data for dichotomous outcomes, we will perform the two following sensitivity analyses on both the primary and secondary outcomes.

- ‘Best-worst-case’ scenario: We will assume that all participants lost to follow-up in the experimental group have survived, had no serious adverse event, had no heart failure, had no stroke, and converted to sinus rhythm and that all those participants lost to follow-up in the control group have not survived, had a serious adverse event, had a heart failure, had a stroke, and did not convert to sinus rhythm.
- ‘Worst-best-case’ scenario: We will assume that all participants lost to follow-up in the experimental group have not survived, had a serious adverse event, had a heart failure, had a stroke, and did not convert to sinus rhythm and that all those participants lost to follow-up in the control group have survived, had no serious adverse event, had no heart failure, had no stroke, and converted to sinus rhythm.

We will present results of both scenarios in our review.

When analysing quality of life or heart rate control, a ‘beneficial outcome’ will be the group mean plus two standard deviations (SDs) (we will secondly use one SD in another sensitivity analysis) of the group mean and a ‘harmful outcome’ will be the group mean minus two SDs (we will secondly use one SD in another sensitivity analysis) of the group mean [81].

To assess the potential impact of missing SDs for continuous outcomes, we will perform the following sensitivity analysis.

- Where SDs are missing and it is not possible to calculate them, we will impute SDs from trials with similar populations and low risk of bias. If we find no such trials, we will impute SDs from trials with a similar population.

We will present results of this scenario in our review.

Other post hoc sensitivity analyses might be warranted if unexpected clinical or statistical heterogeneity is identified during the analysis of the review results [81].

#### ‘Summary of Findings’ table

We will create a ‘Summary of Findings’ table using each of the prespecified primary and secondary outcomes (all-cause mortality, serious adverse event, quality of life, stroke, heart failure, heart rate control, and conversion to sinus rhythm). We will use the five GRADE considerations (bias risk of the trials, consistency of effect, imprecision, indirectness, and publication bias) to assess the quality of a body of evidence as it relates to the studies which contribute data to the meta-analyses for the prespecified outcomes [81, 97–99]. We will use methods and recommendations described in Chapter 8 (Section 8.5) and Chapter 12 of the Cochrane Handbook for Systematic Reviews of Interventions [62] using GRADEpro software. We will justify all decisions to downgrade the quality of studies using footnotes, and we will make comments to aid the reader’s understanding of the review where necessary. Firstly, we will present our results in the ‘Summary of Findings’ table based on the results from the trials with low risk of bias; secondly, we will present the results based on all trials.

## Discussion

Observational evidence has indicated that digoxin might increase the risk of serious adverse events in patients with atrial fibrillation—on the other hand, digoxin is a recommended and commonly used intervention. Moreover, clinical guidelines postulate that no head-to-head randomised clinical trials of digoxin versus other medical interventions in relation to heart rate control in patients with atrial fibrillation or atrial flutter exists—still, we can identify several trials on the topic by searching PubMed. The present systematic review aims at comparing digoxin with placebo, no intervention, or with other medical interventions in patients with atrial fibrillation and atrial flutter. The outcomes will be all-cause mortality, serious adverse events, quality of life, stroke, heart failure, heart rate control, and conversion to sinus rhythm. Due to the millions affected around the World, there is an urgent need of this systematic review.

This protocol has a number of strengths. The predefined methodology is based on the Cochrane Handbook for Systematic Reviews of Interventions [62], the eight-step assessment suggested by Jakobsen et al. [81], Trial Sequential Analysis [64], and GRADE assessment [97–99]. Hence, this protocol takes into account both risks of systematic errors and risk of random errors.

Our protocol also has a number of limitations. The primary limitation is that we in one of our analyses as control intervention intend to meta-analyse digoxin compared with all other types of medical interventions for atrial fibrillation and atrial flutter. Hence, if the different types of control interventions have different effects compared with digoxin, the statistical heterogeneity might be considerable and meta-analysis of all trials in one analysis might not be valid. The reason for this meta-analysis of different control interventions is that we do not expect to identify many relevant randomised clinical trials and we want to increase the statistical power. We have predefined a number of subgroup analyses and sensitivity analyses to assess if the effects differ between trials using different types of control interventions, and we will thoroughly consider if it is valid to perform meta-analysis of all included trials. A further limitation is the large number of subgroup analyses which increases the risk of a type 1 error. We have adjusted our thresholds for significance according to the number of primary outcomes, but we have also included multiple subgroup analyses and assess all outcomes at two time points. The large risk of type 1 error will be taken into account when interpreting the results of the review.

## Additional files

Additional file 1: PRISMA-P 2015 checklist. (DOCX 30 kb)

Additional file 2: The preliminary search strategy for MEDLINE (Ovid). (PDF 221 kb)

## Abbreviations

AFFIRM: The Atrial Fibrillation Follow-up Investigation of Rhythm Management
CENTRAL: The Cochrane Central Register of Controlled Trials
CFDA: China Food and Drug Administration
CI: Confidence interval
EMA: European Medicines Agency
EMBASE: Excerpta Medica database
GRADE: The Grading of Recommendations Assessment Development, and Evaluation
LILACS: Latin American and Caribbean Health Sciences Literature
MD: Mean difference
MEDLINE: Medical Literature Analysis and Retrieval System Online
PRISMA: Preferred Reporting Items for Systematic Reviews and Meta-Analyses
PRISMA-P: Preferred Reporting Items for Systematic Reviews and Meta-Analysis Protocols
PROSPERO: International Prospective Register of Systematic Reviews
RR: Risk ratio
SMD: Standardised mean difference
TRIP: Turning Research into Practice
WHO: World Health Organization
